# Shoulder surface temperature and bone scintigraphy findings in patients with rotator cuff tears

**DOI:** 10.3109/03009734.2010.545150

**Published:** 2011-04-12

**Authors:** Yoichi Koike, Hirotaka Sano, Takeshi Kinjyo, Itaru Imamura, Onuma Masahiro, Masako Goto, Masamizu Ooyama, Atushi Kita, Eiji Itoi

**Affiliations:** ^1^Department of Orthopaedic Surgery, Japanese Red Cross Sendai Hospital, Yagiyama Honcyo, Taihaku-Ku, Sendai, Miyagi, Japan; ^2^Department of Orthopaedic Surgery, Tohoku University School of Medicine, 1-1, Seiryo, Aoba-Ku, Sendai, Miyagi, Japan

**Keywords:** Bone scintigraphy, complex regional pain syndrome (CRPS), complications, rotator cuff tear, skin temperature

## Abstract

**Background:**

Complex regional pain syndrome (CRPS) is one of the serious complications after surgical treatment of a rotator cuff tear. Both a measurement of body surface temperature and bone scintigraphy have been used as diagnostic tools for the early phase of CRPS.Unfortunately, few studies have been carried out that applied these methods to the patients after rotator cuff repair.

**Purposes:**

To clarify both shoulder surface temperature and bone scintigraphy findings in patients with rotator cuff tears.

**Subjects and methods:**

Subjects comprised patients with unilateral rotator cuff tears (five men and five women, mean age 61 years). For measurements of shoulder surface temperature, a Thermochron was attached to both shoulders. As for bone scintigraphy, intravenous injection of technetium-labelled hydroxymethylenebisphosphonic acid (99mTc-HMDP)was performed, and then images were taken with a gamma camera.

**Results:**

During the measurements, the changes in body surface temperature for the affected and healthy shoulders remained within the standard deviation of the reference group. The intensity of radioisotope (RI) uptake for the affected shoulder joint was significantly increased compared to that for the healthy shoulder joint (*P* < 0.05).

**Conclusion:**

RI uptake is increased in shoulders with rotator cuff tears, whereas shoulder surface temperature shows no differences on the affected and unaffected sides.

## Introduction

Complex regional pain syndrome (CRPS) is a complication that can occur after surgical treatment of a rotator cuff tear (1,2). Primary characteristics of CRPS are abnormal temperature and bone resorption in the afflicted limb (1,3–5). In the clinical setting, abnormal temperature of the afflicted limb was detected by measuring body surface temperature (3,5), and bone resorption was detected by bone scintigraphy in patients with CRPS (3,6).

When body surface temperature measurements and bone scintigraphy are performed on a patient after rotator cuff tear surgery, one should interpret the findings appropriately based on the proper control data. To obtain the base-line data, both measurements of body surface temperature and bone scintigraphy should be carried out before surgery. Base-line data is extremely important to interpret the postoperative data appropriately.

Unfortunately, to date, bone scintigraphy findings on preoperative patients with rotator cuff tears have not been reported yet. Mikayoshi et al. conducted time-dependent measurements over a 24-hour period (7), but there is no report of a follow-up test. Based on these backgrounds, we attempted to clarify the shoulder surface temperature as well as the bone scintigraphy findings in patients with rotator cuff tears.

## Participants and methods

### Patients

Participants were ten patients (five men and five women) who were hospitalized for rotator cuff tear treatment. The mean age was 61 years (range 39–76 years), and the average period of affliction was 6 months (range 3–17 months). Five patients had a history of injury, and four had contractures as complications. All patients complained of pain at rest and nocturnal pain. The average DASH score (8) was 52 points (range 40–75), and the average JOA score (9,10) was 58 points (range 46–70). None of the patients satisfied the CRPS diagnostic criteria established by the Japan Ministry of Health, Labour, and Welfare (11).

On simple X-ray images, six cases showed some osteosclerosis at the greater tuberosity and three cases showed some osteosclerosis of the glenoid. Magnetic resonance imaging demonstrated five full-thickness tears and five partial-thickness tears. Seven tears involved only the supraspinatus tendon, and three involved multiple tendons. Preoperative ultrasound examinations on all patients confirmed that the rotator cuff tears were unilateral.

### Measurements of shoulder surface temperature

Thermochrons (Maxim Integrated Products, Sunnyvale, CA, USA) (surface thermometers with a diameter of 15 mm and built-in temperature sensor, memory, and battery) were used for measurements (12). The measurement method has been reported previously (2).

Briefly, thermochrons were attached 5 cm below the anterolateral end of the acromion on both sides. Insulating tape (Nitoms Inc., Tokyo, Japan) was applied to the surface of the thermochrons, and an adhesive sheet (Perme-Roll, Nitto medical Co., Osaka, Japan) further covered the taped surface. Anticipating when the patients would be sleeping, the measurement period was set from 21.00 until 07.00 the next morning. The time between measurements was 15 minutes, resulting in 40 measurements taken during the night. After completing the measurements, all thermochrons were removed, and the data were collected.

For the reference group, we used previously reported measurement data from a group of ten individuals without shoulder problems (20 limbs) (2). The reference group included four men and six women (mean age 54 years). There were five cases of lower leg fracture, three cases of foot fracture, and two cases of hallux valgus.

### Bone scintigraphy

Intravenous injection of 740 MBq technetium-labelled hydroxymethylenebisphosphonic acid (99mTc-HMDP) was performed slowly, and images were taken 3 hours later with a gamma camera (Symbia T2, Siemens AG, Berlin, Germany). The shoulder, elbow, and wrist joints on both sides of a forward-facing image of the entire body were established as regions of interest (ROIs). The level of radioisotope (RI) uptake in each ROI was reported as a relative intensity normalized against the background uptake level.

Ten patients (five men and five women; mean age 61 years) who had no complaints regarding the upper limbs (20 limbs) were among those for whom bone scintigraphy had been carried out at this hospital during 2009 and were used as the reference group. Diagnoses at the time of bone scintigraphy testing for the reference group were: one case of pubic fracture, two cases of lumbar vertebral compression fracture, one case of septic arthritis of the ankle joint, three cases of ankle osteoarthritis, and three cases of femoral head necrosis.

### Statistics

We compared body surface temperatures using means for three groups: the affected side of the affected group, the healthy side of the affected group, and the reference group. In the reference group, both shoulders of ten patients (20 shoulders) were pooled into one group. This is because it has already been reported that there is no statistically significant difference in the body surface temperature between upper limbs in healthy individuals (2).

RI uptake intensities in the shoulder, elbow, and wrist joints were compared between the affected and healthy sides in the rotator cuff tear group. In the reference group, the left and right sides were compared for each joint. PASW Statistics 18 (SPSS, Chicago, IL, USA) was the statistical software used for conducting paired sample *t* tests. The significance criterion was *P* < 0.05.

### Ethics

The protocol of this study was approved by the ethics board of the first author's institute (Registration number R1000245, approved on 20 June 2009) and was conducted in accordance with the Declaration of Helsinki. All patients gave informed consent to participate in this study.

## Results

**Figure 1.**
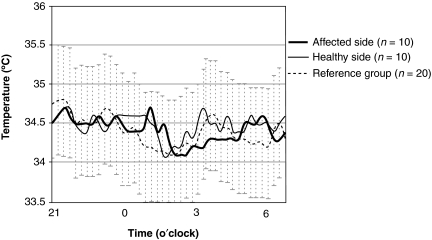
Time-dependent changes in the mean shoulder surface temperature.The changes in body surface temperature for the affected (bold line) and healthy shoulders (thin line) remained within the standard deviation (SD) of the reference group (dotted line with SD bars).

Time-dependent changes in the mean body surface temperature of the affected group (ten shoulders) are shown in Figure 1. From 21.00 to 03.00, the temperature slowly decreased and reached a minimum of 34.1°C at 02.00. Thereafter, the temperature increased slowly until 07.00. During this time, the changes in shoulder surface temperature for the affected and healthy sides remained within the standard deviation of the reference group. In comparing mean temperatures, there were no statistically significant differences among the three groups. The mean (standard deviation) shoulder surface temperature in the affected side, the healthy side, and the reference group were 34.4 (0.2) °C, 34.5 (0.2) °C, and 34.4 (0.2) °C, respectively. None of the groups represented statistical significant differences.

**Figure 2.**
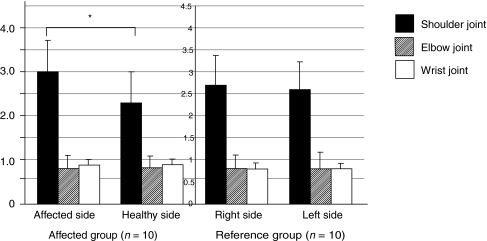
The intensity of radioisotope(RI) uptake. The intensity of RI uptake for the affected shoulder joint is significantly increased compared to that for the healthy shoulder joint. In elbow and wrist joints, there are no significant differences in RI uptake intensity between the affected and healthy sides. In the reference group, no significant left–right differences are seen for the shoulder, elbow, and wrist joints. (*Affected side 3.0 ± 1.0 versus healthy side 2.3 ± 0.7; *P* < 0.05).

In the bone scintigrams, we observed increased RI uptake in the affected shoulder joint in nine cases (90%). The intensity of RI uptake for the affected shoulder joint was significantly increased compared to that for the healthy shoulder joint. The mean (standard deviation) intensity of RI uptake in the affected side and the healthy side were 3.0 (1.0) and 2.3 (0.7), respectively (*P* < 0.05; Figure 2). On the other hand, in elbow and wrist joints, there were no significant differences in RI uptake intensity between the affected and healthy sides. Moreover, no significant differences were found between right and left sides in the reference group for shoulder, elbow, or wrist joints.

### Case presentation

#### Case 1

**Figure 3.**
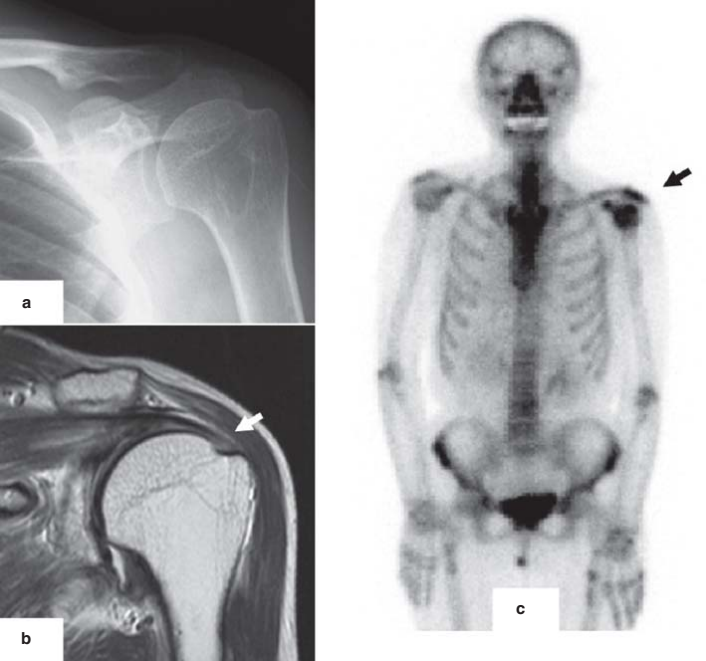
Case 1: A 71-year-old male with a rotator cuff tear in the left shoulder. No obvious changes of osteoarthritis are observed on plain X-rays (A). A full-thickness rotator cuff tear is observed on the coronal T2 WI MRI (B, arrow). An increase of RI uptake in the left shoulder joint is observed in the bone scintigram (C, arrow). However, there are no left–right differences in RI uptake for elbow or wrist joints.

A 71-year-old male was injured after falling on a snow-covered path and hitting his left elbow 6 months earlier. He noticed pain in the left shoulder after the injury, and became aware of nocturnal pain in the left shoulder 4 months after the injury. Physical examination revealed a limited range of motion and muscle weakness in abduction and external rotation of the left shoulder joint. There were positive signs of impingement (13), and a block test (14). Preoperative JOA and DASH scores were 60 out of 100 and 45, respectively. No obvious changes of osteoarthritis were observed on plain X-rays (Figure 3A). A full-thickness rotator cuff tear was observed on MRI (Figure 3B, arrow). An increase of RI uptake in the left shoulder joint was observed in the bone scintigram (Figure 3C, arrow). However, there were no remarkable left–right differences in the RI uptake for elbow or wrist joints.

#### Case 2

**Figure 4.**
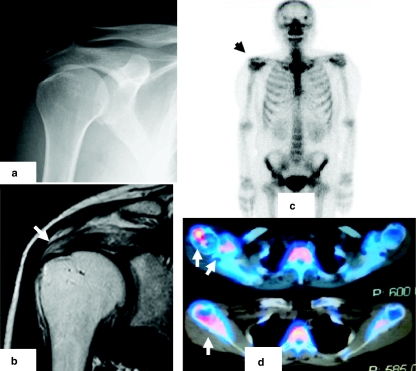
Case 2: A 51-year-old male with a rotator cuff tear in the right shoulder. No obvious osteoarthritis is observed in X-rays (A). A full-thickness rotator cuff tear was observed on the coronal T2 WI MRI (B, arrow). An increase of RI uptake in the left shoulder joint is observed in the bone scintigram (C, arrow). In SPECT images, RI uptakes are evident in the greater tuberosity of the humerus, the coracoid process of the scapula, and the glenohumeral joint (D, arrows).

A 51-year-old male incurred injury 8 months earlier after falling over while riding a motorcycle. He experienced pain from the right shoulder to the forearm after the injury and became aware of nocturnal pain in the right shoulder 6 months after the injury. Physical examination revealed a limited range of motion in abduction, external rotation, and extension of the right shoulder joint. Preoperative JOA and DASH scores were 62 out of 100 and 50, respectively. No obvious arthrotic changes were seen on plain X-rays. A full-thickness rotator cuff tear was observed on MRI. An increase of RI uptake in the right shoulder joint was observed in the bone scintigram. However, there were no left–right differences in RI uptake for elbow or wrist joints. In SPECT (single photon emission computed tomography) imaging carried out at the same time, RI uptake was observed in the greater tuberosity of the humerus, the coracoid process of the scapula, and the glenohumeral joint (Figure 4).

## Discussion

The shoulder surface temperature in a healthy human changes over time and is influenced by daily fluctuations in core body temperature (15,16). Multiple factors, including heat generation associated with muscle contraction, have been reported as external factors affecting body surface temperature (17,18). Changes in body surface temperature occurring in each of the upper limbs could give rise to left–right differences in temperature. However, when multiple measurements are taken across time and means are then compared, no left–right differences are observed (2).

Abnormal shoulder surface temperatures are triggered by various shoulder joint diseases. In adhesive capsulitis and CRPS, the surface temperature of the afflicted shoulder is significantly reduced compared to the healthy side (19,20). In the present study, there was no significant difference between the surface temperature of the affected shoulder compared to that of the healthy shoulder or in the reference group. These results were consistent with those reported previously by Miyakoshi et al. (7), who reported that the body surface temperature of a shoulder with a rotator cuff tear was not different from that of a healthy shoulder. From the clinical point of view, the results of the present study could be interpreted as follows: if a temperature abnormality is observed after rotator cuff surgery, the onset of the temperature abnormality is probably after the surgery.

Bone scintigraphy excels in detecting bone resorption associated with fractures, bone tumours, osteomyelitis, disuse atrophy, and other conditions (6). Increased RI uptake in the cancellous bone of the afflicted limb is a characteristic feature of CRPS, and early bone resorption can be objectively detected using scintigraphy (3).

The increased uptake that occurs in the shoulder joint has previously been observed in patients with adhesive capsulitis (21). Interestingly, it has been reported that the cause of adhesive capsulitis is related to the incomplete onset of CRPS (22,23). Waldburger et al. speculated that the increased RI uptake that occurs in adhesive capsulitis reflected the onset of CRPS (21). It is possible that the increased RI uptake in patients with a rotator cuff tear in the present study also reflects the latent onset of CRPS. Alternatively, it is possible that arthritis, osteophyte formation, or treatments prior to RI examination affected this. Clinically, this increase in RI uptake might be seen in patients after shoulder joint surgery. In such cases, the increase of RI uptake might be misdiagnosed as CRPS onset after surgery. However, sufficient attention should be paid to whether increased RI uptake could be observed in the joint preoperatively.

In summary, we reported findings on shoulder surface temperature and bone scintigraphy in patients with rotator cuff tears. The surface temperature of shoulders with rotator cuff tears was not different from that of the contralateral normal shoulders or shoulders in the healthy reference group. This suggests that if a temperature abnormality is observed after rotator cuff surgery, the onset is likely a postoperative one. Bone scintigraphy shows an increased RI uptake in shoulders with rotator cuff tears. Careful attention should be paid when interpreting RI images, so as not to misdiagnose that these findings were caused by CRPS in patients after rotator cuff surgery.
